# Correlations in the degeneracy of structurally controllable topologies for networks

**DOI:** 10.1038/srep46251

**Published:** 2017-04-12

**Authors:** Colin Campbell, Steven Aucott, Justin Ruths, Derek Ruths, Katriona Shea, Réka Albert

**Affiliations:** 1 Department of Physics, Washington College, Chestertown, MD 21620, USA; 2 Department of Mechanical Engineering, University of Texas at Dallas, Richardson, TX 75080, USA; 3 Department of Computer Science, McGill University, Montreal, Quebec H3A 2A7, Canada; 4 Department of Biology, Pennsylvania State University, University Park, PA 16802, USA; 5 Department of Physics, Pennsylvania State University, University Park, PA 16802, USA.

## Abstract

Many dynamic systems display complex emergent phenomena. By directly controlling a subset of system components (nodes) via external intervention it is possible to indirectly control every other component in the system. When the system is linear or can be approximated sufficiently well by a linear model, methods exist to identify the number and connectivity of a minimum set of external inputs (constituting a so-called minimal control topology, or MCT). In general, many MCTs exist for a given network; here we characterize a broad ensemble of empirical networks in terms of the fraction of nodes and edges that are *always, sometimes*, or *never* a part of an MCT. We study the relationships between the measures, and apply the methodology to the T-LGL leukemia signaling network as a case study. We show that the properties introduced in this report can be used to predict key components of biological networks, with potentially broad applications to network medicine.

A system of interacting components can be represented by a network, where system components are represented by *nodes* (or *vertices*) and their interactions are represented by directed *edges* (or *links*) between nodes. The dynamical behavior of the system is constrained by the topology of the network, but, because an edge generally indicates the existence of a regulatory relationship without specifying its nature, network topology is necessarily an incomplete representation of the system’s dynamical behavior. For instance, the dynamic relationship represented by a node *A* being regulated by two nodes *B* and *C* may be well-approximated by a truth table (e.g., the future state of *A* is given by *B* AND *C*, where the node state variables are binary, 0 or 1) or by an arbitrarily complex function of *B* and *C* (e.g., *dA/dt* = *B*^*C*^, where the node state variables are real numbers).

Whatever the dynamical relationships, a common goal is to efficiently influence the behavior of the constituent complex system. For example, manipulating biological systems can potentially lead to the development of effective therapies for disease^1^,^2^,^3^,^4^,^5^, ecological management may preserve vital ecosystem services^6^,^7^,^8^,^9^,^10^, and technological systems such as the power grid must be carefully designed and managed to provide functional and efficient services to consumers^11^,^12^,^13^,^14^,^15^. The field of network science aims to identify the extent to which the network structure alone can be used to study the dynamics, and more recently the control, of systems. In part because such analysis aims to identify model-agnostic properties and in part because it can provide informative first-order effects, it is common to model the evolution of systems by linear dynamics of the form


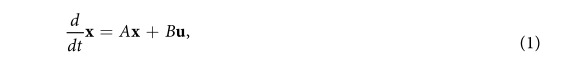


where **x** is a vector of the nodes in the network, *A* is the *adjacency matrix* that encodes the interactions in the network topology (including, for instance, diagonal terms corresponding to the free decay of a node state variable), and the term *B***u** encodes external, user-controlled signals fed into a subset of so-called directly-controlled nodes, i.e., the dynamics of these specific nodes are directly affected by these external signals. Many systems, of course, are inherently nonlinear and are at best approximately linear within a local region; assuming linear dynamics of the form of equation (1) in these cases is an extremely coarse approximation^16^,^17^,^18^, thought note that linearizing a nonlinear system is in some cases a sufficient condition for local controllability^19^. Nonetheless, the study of linear dynamics is important not only for systems that *are* linear, but also as a comparative framework for nonlinear dynamics: a system is nonlinear to the extent that it departs from linear expectations. The extent to which our results apply to nonlinear systems will depend on the form of the nonlinearity and the region of interest. While the networks we study behave according to a variety of models, we use their interaction networks to provide realistic structures for us to study the potential degeneracies of control properties.

In this case it is possible, with a sufficient number and placement of directly-controlled nodes and appropriate choices of time-varying control signals, to drive the system to any desired state in finite time^20^,^21^,^22^,^23^. In principle one wishes to control the dynamics of a network with as few interventions as possible; minimizing the number of controls amounts to finding the *maximum matching* on the network (see Methods). Recent work has addressed the relationship between network topology and the properties of the directly-controlled nodes. For instance, the directly-controlled nodes are either source nodes or arise due to a *dilation*, where a node has more than one outgoing edge^23^. By classifying dilations as *external* (if the outgoing edges point to sink nodes) or *internal* (otherwise), one can determine the fraction of controls in each of these categories (respectively denoted *η*_*s*_, *η*_*e*_, and *η*_*i*_). These parameters constitute the *control profile* of a network. Empirical networks tend to be dominated by one of the three control profile parameters; diverse mechanisms exist by which synthetic networks may be generated with the same properties^23^,^24^,^25^,^26^.

While the control profile offers insight into the types of directly-controlled nodes in terms of their topological location, it offers no direct insight into the *degeneracy* of control: there are generally many solutions to the maximum matching of a network. In other words, many combinations of nodes may be chosen for direct control. Furthermore, determining the maximum matching involves not only a set of directly-controlled nodes, but also a set of *matched edges*, which constitute *control signal paths* (see Fig. 1, Methods). It is therefore of interest to assess the importance of both nodes and edges in the set of all of the so-called *minimal control topologies* (MCTs). Specifically, here we wish to determine if a node is *always, sometimes*, or *never* directly controlled among all maximum matchings (i.e., across all MCTs). Similarly, we wish to determine if an edge is *always, sometimes*, or *never* on a control signal path.

Our contributions in this report are three-fold. First, we leverage the methodology of Jia *et al*.^21^ to characterize a new network-level statistic capturing the fraction of nodes that are always, sometimes, or never directly controlled (*υ*_*a*_, *υ*_*s*_, and *υ*_*n*_, respectively). We then develop a new method using a similar approach to assign a network-level statistic capturing the fraction of edges that always, sometimes, or never belong to the set of matched edges (fractions *ε*_*a*_, *ε*_*s*_, and *ε*_*n*_, respectively). These new quantities echo the spirit of the control profile, which is a unique network-level statistic that captures the fraction *of controls* due to different functional structures. In contrast, these new quantities capture the fraction *of nodes and edges* that participate in the process of control signal dissemination through the network in varying ways. Thus the third contribution of this work is to provide insight into the connections between the functional classification of the control configuration (given by the control profile) and the degeneracy of the control configuration allowed by the network structure (captured by the statistics proposed in this report). Notably, we show that the existence of many internal dilations correlates with many edges *never* existing on a control path, suggesting that internal dilations tend to restrict the flexibility with which control signals propagate through a network. Finally, we apply the methodology of this report to the T-LGL leukemia signaling network and show broad agreement between the metrics introduced herein and existing experimental, computational, and analytical work that has identified nodes whose control play a pivotal role in the behavior of the network. While (as noted above) it is known that the study of structural controllability has some applications to systems characterized by nonlinear dynamics, the above-summarized results provide additional evidence suggesting that the methodology described in this report may be used in other biological networks to predict network components essential for control, with potentially broad applications to network medicine.

## Results

We analyze 58 empirical networks (see Table S1) and determine their distribution in parameter space for the control profile, node-based degeneracy, and edge-based degeneracy measures (Fig. 2a–c). As previously reported, the control profile of any one empirical network tends to be dominated by one of the control profile parameters^23^. In contrast, the node-based degeneracy measures indicate that *ν*_*a*_ ≪ 1 for most networks, that is, few networks have a significant fraction of nodes that are always directly controlled (in agreement with the observation that most networks have relatively few directly-controlled nodes). This observation applies also to the edge-based degeneracy measures; *ε*_*a*_ ≪ 1. However, while networks are well-dispersed between *ν*_*s*_ and *ν*_*n*_, most networks are skewed toward *ε*_*s*_, meaning that while some networks have many nodes that are never directly controlled, few networks have many edges that are never on a control signal path.

We are also interested in the relationships between these measures. In Fig. 2(d–f) we show the distributions of degeneracy measures separately for networks where each of the control profile measures is largest (e.g., *η*_*s*_ > *η*_*e*_, *η*_*i*_). We find, for instance, that *η*_*s*_-dominated networks tend to be *ν*_*n*_- and *ε*_*s*_-dominated. In other words, networks where many of the directly-controlled nodes are source nodes tend to have many nodes that are never directly controlled, and many edges that can be (but are not necessarily) on control signal paths. In contrast, *η*_*e*_-dominated networks tend to be *ν*_*s*_- and *ε*_*s*_-dominated, meaning that networks with an abundance of sink nodes tend to have significant flexibility both in choice of directly-controlled nodes and control signal paths. Finally, *η*_*i*_-dominated networks tend to be *ε*_*s*_-dominated (though less so than in the case of either *η*_*s*_-dominance or *η*_*e*_-dominance) and distributed between *ν*_*s*_ and *ν*_*n*_. Thus, networks with many internal dilations may or may not have flexibility in terms of choice of directly-controlled nodes, and tend to have at least a moderate degree of flexibility in control signal paths.

We perform a pairwise Spearman correlation analysis between each of the 9 control-related parameters considered here and with basic network properties (Table 1). Unsurprisingly, there is a strong negative correlation between *ε*_*a*_ and each of the average node degree, average clustering coefficient, and network transitivity (−0.79, −0.52, and −0.58, respectively): more connections per node and/or an increased frequency of closed triads afford greater flexibility in assigning control signal paths. Interestingly, these same properties are negatively correlated with *ν*_*s*_ and positively correlated with *ν*_*n*_: a richer local structure constrains nodal participation (as directly controlled nodes) in MCTs. Furthermore, the number of nodes and edges exhibit weak negative correlation with *ν*_*n*_ and *ε*_*n*_, indicating that larger networks are more likely to access more nodes and edges in at least some MCTs. Measures within a set tend to be negatively correlated with one another, with the notable exception of *ε*_*a*_and *ε*_*n*_ (0.29). Some powerful trends exist between sets, as well, as suggested by Fig. 2: *η*_*s*_ is correlated with *ν*_*a*_ (0.8), *η*_*i*_ is correlated with *ε*_*n*_ (0.74), and *η*_*e*_ is correlated with *ν*_*s*_ (0.68).

To validate the utility of these measures, we consider the dynamic model of survival signaling network relevant to T-LGL leukemia^2^,^27^. In this disease a fraction of white blood cells activated in response to a stimulus escape the process of activation induced cell death, survive, and after a while start attacking healthy cells. The dynamics of this network are defined by Boolean functions, from which a topological network can be extracted such that *A*->*B* if node *A* exists in the update function for node *B*. In the T-LGL network the node representing apoptosis (i.e., programmed cell death) is of particular interest. Its OFF state, together with the deregulation (abnormally high or low activity) of a subset of nodes, indicates the abnormal, leukemic state. Conversely, if in a leukemic cell the state of apoptosis cell changes from OFF to ON, the cell is committed to the process of cell death. Existing work has identified the minimal set of nodes whose sustained expression can lead to the leukemic state. This set consists of three source nodes: the initial stimulus, as well as the external molecules platelet-derived growth factor (PDGF) and interleukin (IL) 15, both of which were experimentally observed to be over-abundant in the blood of T-LGL leukemia patients^27^. Prior work has also identified nodes whose direct control can lead to apoptosis of leukemic cells, despite the continued presence of these source nodes. Control of any one of 18 nodes (of 57 total) leads to apoptosis according to at least two of the following three types of evidence: experimental verification^27^, simulation of Boolean dynamics^2^, and analysis of the topology of the network once it has been expanded to topologically encode the Boolean rules^28^.

The extent to which we expect the present metrics to agree with prior work is mitigated to some extent by the scope of the methodologies: in most prior work the quantity of interest is the state of a *single* node (apoptosis), whereas structural controllability seeks to achieve a desired state for *every* node in the network. Furthermore, the methodology used here assumes dynamics that obey equation (1), which is quite different from the Boolean framework used in the prior work being considered here. Therefore, a conservative expectation is that the two methodologies do not contradict one another. Specifically, assuming that the target state reflects induced apoptosis of a leukemic cell, we expect that the three source nodes necessary for the leukemic state are always directly controlled and the 18 apoptosis-inducing nodes should, at minimum, sometimes be directly controlled and/or be connected to an edge that is always on a control path.

We verify that this is the case: all three source nodes are always directly controlled, and 15 of the 18 key nodes have at least one incoming or outgoing “always” edge (indicating that they take part in a critical signaling pathway in terms of control) and/or are sometimes directly controlled (indicating that in some cases system control may require direct control of these nodes). Furthermore, the remaining three key nodes are connected to at least 4 “sometimes” edges (indicating flexibility in the manner in which control signals are routed through these nodes). Indeed, despite the different methodological frameworks, the agreement is rather strong: all 18 nodes are connected to more “always” and/or more “sometimes” edges than expected by random chance, and only 2 are connected to more “never” edges than expected by random chance (for more details, see the Supplementary Information).

## Discussion

**Figure 1:**
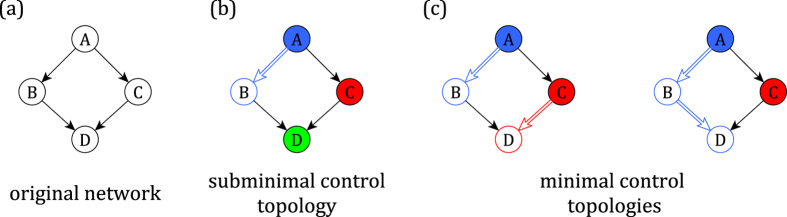
Control topologies in networks with linear, non-dissipative dynamics. (**a**) A simple directed network. We omit cycles from this example because in this framework they are inherently self-regulatory and their control follows immediately once the remainder of the network has been controlled^23^. (**b**) In a control topology, every node is either directly controlled (colored nodes) or indirectly controlled (white nodes with colored outlines). Indirect control is achieved by placing nodes on a path originating at a directly controlled node (white edges with colored outlines). Importantly, in this framework every node can control at most one of its downstream neighbors and every pair of such paths are necessarily node-disjoint. (**c**) A control topology is minimal if it minimizes the number of controls. In this example node A must be directly controlled (it has no upstream nodes through which a control path may be routed) and either node B or node C must be directly controlled because node A can control at most one of its downstream neighbors.

**Figure 2:**
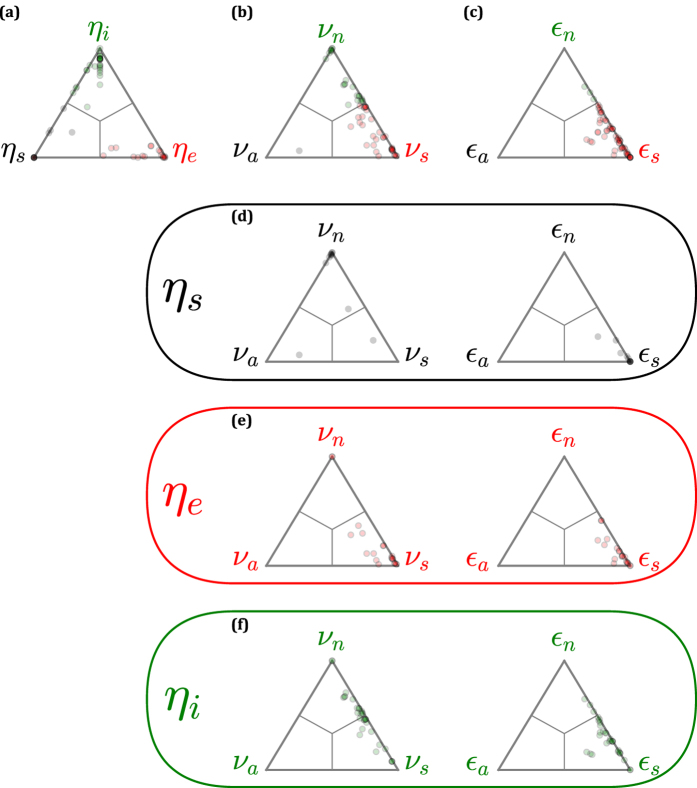
Distributions of empirical networks according to three sets of control measures. Each set includes three measures that sum to 1 for a given network. The distributions are shown on ternary plots, where a network at the center corresponds to a set of values (1/3, 1/3, 1/3) and a network at a corner corresponds to e.g. (1, 0, 0). Networks are represented with colored according to their maximal parameter. Interior lines indicate the regions where each parameter is largest. **(a)** The control profile of Ruths & Ruths^23^. Directly-controlled nodes are either source nodes (*η*_*s*_), arise due to internal dilations (*η*_*i*_), or arise due to external dilations (*η*_*e*_). **(b)** The fraction of nodes that are always (*ν*_*a*_), sometimes (*ν*_*s*_), or never (*ν*_*n*_) directly controlled, when considering all control schemes that minimize the number of controls. **(c)** The fraction of edges that are always (*ε*_*a*_), sometimes (*ε*_*s*_), or never (*ε*_*n*_) on a control signal path, when considering all control schemes that minimize the number of controls. **(d**–**f)** The degeneracy measures applied independently to the cases where *η*_*s*_*, η*_*e*_, and *η*_*i*_ are the dominant parameter in the control profile. Each plot is uniformly colored according to the corresponding dominant control profile parameter (as labeled on the left of the panel). The formatting of each plot otherwise follows (**a**–**c**).

Effectively influencing the behavior of complex interacting systems is a broad, multi-disciplinary goal. Accordingly, there is significant interest in discovering general techniques by which the dynamics of systems from different domains (e.g., technological and biological) may be guided by external intervention. We here consider systems that obey the linear dynamics of equation (1), where it has been shown that complete control is possible by feeding external control signals into a subset of the system components^20^,^21^,^22^,^23^. These directly-controlled nodes are chosen such that every other node in the network is reached via non-overlapping paths originating at the directly-controlled nodes (see Fig. 1). The directly-controlled nodes and these control paths together constitute a *control topology*; a minimal *control topology* (MCT) is one in which the number of inputs is minimized.

In this report we consider the degeneracy of minimal control (i.e. the extent to which different minimal control topologies exist for a given network) in linear systems. Specifically, we characterize every system component (node) and interaction (edge) as being *always, sometimes,* or *never* on a MCT (the fraction of all nodes in these categories are respectively represented by the parameters *ν*_*a*_, *ν*_*s*_, and *ν*_*n*_ for nodes and *ε*_*a*_, *ε*_*s*_, and *ε*_*n*_ for edges). We study a broad selection of empirical networks and find that they are generally distributed between *ν*_*s*_and *ν*_*n*_ while *ν*_*a*_tends to be small. While we can unambiguously state that nodes are always directly controlled only if they are source nodes^23^, in all but the simplest networks more precise statements require analysis of the maximum-matching problem and/or perturbing a MCT via a breadth-first search (see Methods). However, the flexibility of control in this framework is reflected by the typically high values of *ε*_*s*_, suggesting that there are generally many ways for control signals to propagate through a network, even if there is relatively little flexibility in the choice of nodes to be directly controlled.

We consider these measures against the fraction of controls that are source nodes, sink nodes, and internal nodes (*η*_*s,*_, *η*_*e*_ and *η*_*i*_, respectively), quantities which are fixed for a given network^23^. The fact that *η*_*s*_ is positively correlated with *ν*_*a*_ follows from their definitions: *η*_*s*_ is the fraction of *directly-controlled nodes* that are source nodes, and *ν*_*a*_ is the fraction of *all nodes* that are source nodes (see Methods). The fact that *η*_*i*_ is positively correlated with *ε*_*n*_indicates the existence of some rigidity in control signal paths cases where most of the directly-controlled nodes are neither sources nor sinks. In contrast, the correlation between *η*_*e*_ and *ν*_*s*_ suggests flexibility when most of the control nodes are sink nodes. In other words, there is flexibility in choosing *which* sink nodes are directly controlled and which are not, likely in part because there are multiple paths from source nodes to different sink nodes. It is also interesting to note that the correlations between node-based degeneracy measures and edge-based degeneracy measures tends to be weak (with the exception of *ν*_*a*_and *ε*_*a*_, the correlation magnitudes are uniformly below 0.4), indicating a relative disconnect between the node-based and edge-based degeneracy measures considered in this report.

While an interesting topic from a strictly theoretical standpoint, characterizing control degeneracy also has significant practical implications. In a biological system, for instance, a particular group of signaling molecules may be implicated in many theoretically viable control strategies. This, in turn, could incentivize the development of (e.g., pharmacological) techniques to influence the molecules in question. Indeed, we have shown broad agreement between the techniques developed here and existing work concerning the dynamics of the T-LGL leukemia signaling network, and the techniques described herein could be used to identify potential candidates for regulatory control in other biological networks. In an ecological system, the abundance of a particular group (or groups) of species, for example invasive or endangered species, may be controlled to initiate a cascade of changes in the abundances of other species^6^,^7^,^8^,^29^,^30^,^31^,^32^. Species implicated in many viable control strategies under an appropriate modeling framework may, therefore, be prime candidates for direct manipulation to effectively manage ecological communities. Regardless of context, in cases where the nature of any nonlinearity is unknown, the methods developed here may provide insight into which components are essential for control.

We observe in this study that there exist meaningful correlations between the degeneracy of the control topology (directly controlled nodes and matched edges) and the functional divisions offered by the control profile. While aggregated statistics of network controllability have offered fruitful insights in the past, moving forward – to understand more precisely how network topology is related to network control – will require knowledge about all the possible control paths that can be used to control a network. Ultimately we aim to provide a clear mapping between the structure of the network and the ability we have to control such a system. Here we have provided a new dimension such that we can use the types of degeneracy exhibited by the control topology along with the dominance of certain types of control structures (given by the control profile) to triangulate more informed inferences on the network structures that are most important for network control.

A tempting avenue for future work is the development of procedures that identify the *fraction* of control paths that contain a given node or edge. This information would allow the categorical analysis considered in this report to be complemented by analysis on a continuum: nodes in *V*_*a*_ are in 100% of all control paths, nodes in *V*_*n*_ are in 0%, and nodes in *V*_*s*_ are somewhere in between (and similarly for edges). Studying the properties that drive nodes and edges to have comparatively high or low participation in control paths promises to enhance our understanding of the relationship between the structure and controllability of complex systems. In addition, we note that the diverse selection of empirical networks evaluated in this study offers insight into network structures that are independent of context. While this follows related work and avoids sample bias that arises when considering traditional generative models^23^,^26^,^33^, taking a similar approach as this study, but focused upon a particular empirical context (e.g., cellular signaling networks, ecological networks) may offer network-specific insight.

## Methods

### Control Topology

We define a control topology as a set of directly-controlled nodes, *N*_*d*_, and the corresponding control signal paths that yield indirect control over every other node in the network^20^,^23^. A control topology is minimal if it additionally minimizes |*N*_*d*_|. Prior work has generally assessed the properties of a single minimal control topology (MCT) for a given network; a MCT is often obtained via the Hopcroft-Karp algorithm^20^,^23^,^34^,^35^. In Fig. 1 we show several control topologies for a simple network.

### Node-based assessment of MCT degeneracy

Because many MCTs generally exist for all but the simplest networks, we wish to characterize the nodes in a network according to the frequency with which they are directly controlled in a MCT. Specifically, a node is *always, sometimes*, or *never* directly controlled in a MCT; we denote the set of nodes in these categories as *V*_*a*_, *V*_*s*_, and *V*_*n*_, respectively. Similarly, we denote the size of each set, normalized by the total number of nodes in the network, as *ν*_*a*_, *ν*_*s*_, and *ν*_*n*_.

To categorize the nodes in this way, we adopt the method proposed by Jia *et al*.^21^. Suppose a single MCT has been determined, and consider first the set of directly-controlled nodes *N*_*d*_. Clearly every node *n* ∈ *N*_*d*_ is a member of either *V*_*a*_ or *V*_*s*_. Making this distinction is trivial in light of the fact that the set of source nodes is identical to *V*_*a*_^21^. It follows immediately that the directly-controlled nodes in the MCT that are *not* source nodes are members of *V*_*s*_.

It remains only to consider the nodes *n* ∉ *N*_*d*_. Clearly every such node is a member of either *V*_*s*_ or *V*_*n*_. To determine the membership of one such node *n*_*i*_, we force it to be directly controlled: if |*N*_*d*_| increases as a result, then it immediately follows that no MCT directly controls node *n*_*i*_ and therefore *n*_*i*_ ∈ *V*_*n*_. Otherwise, *n*_*i*_ ∈ *V*_*s*_. We repeat this procedure for all nodes *n* ∉ *N*_*d*_. It is possible to force a node to be directly controlled by perturbing the original MCT with an algorithmic complexity *O(EN*) (see SI).

### Edge-based control classification

Here we are interested in similarly classifying *edges* as *always, sometimes*, or *never* existing on the path of a control signal. We respectively define the sets of nodes in these categories as *E*_*a*_, *E*_*s*_, and *E*_*n*_, and the normalized sizes of these sets as *ε*_*a*_, *ε*_*s*_, and *ε*_*n*_. As in the case of the node-based analysis, we begin by applying the Hopcroft-Karp algorithm to the network in question to determine one MCT. From this MCT we obtain a set of edges on control paths, *L*_*c*_ (e.g., edge A->B in Fig. 1a).

Clearly edges *l* ∈ *L*_*c*_ are members of *E*_*a*_ or *E*_*s*_. Similarly, edges *l* ∉ *L*_*c*_ are members of *E*_*s*_ or *E*_*n*_. In the first case, removing one such edge *l*_*ji*_ (denoting an edge from node *j* to node *i*) and re-evaluating the number of directly-controlled nodes via the Hopcroft-Karp algorithm serves to categorize the node: if |*N*_*d*_| increases, *l*_*ji*_ ∈ *E*_*a*_; otherwise *l*_*ji*_ ∈ *E*_*s*_. In the second case we may wish to force an edge *l*_*ji*_ ∉ *L*_*c*_ to be on a control path and similarly re-apply the Hopcroft-Karp algorithm; however, no simple modification to the network guarantees that the Hopcroft-Karp algorithm will force *l*_*ji*_ ∈ *L*_*c*_.

We therefore develop alternatives for both of the above cases; the approach has a complexity of *O*(E^2^) (see SI).

## Additional Information

**How to cite this article**: Campbell, C. *et al*. Correlations in the degeneracy of structurally controllable topologies for networks. *Sci. Rep.* **7**, 46251; doi: 10.1038/srep46251 (2017).

**Publisher's note:** Springer Nature remains neutral with regard to jurisdictional claims in published maps and institutional affiliations.

## Supplementary Material

Supplementary Information

## Figures and Tables

**Table 1:**
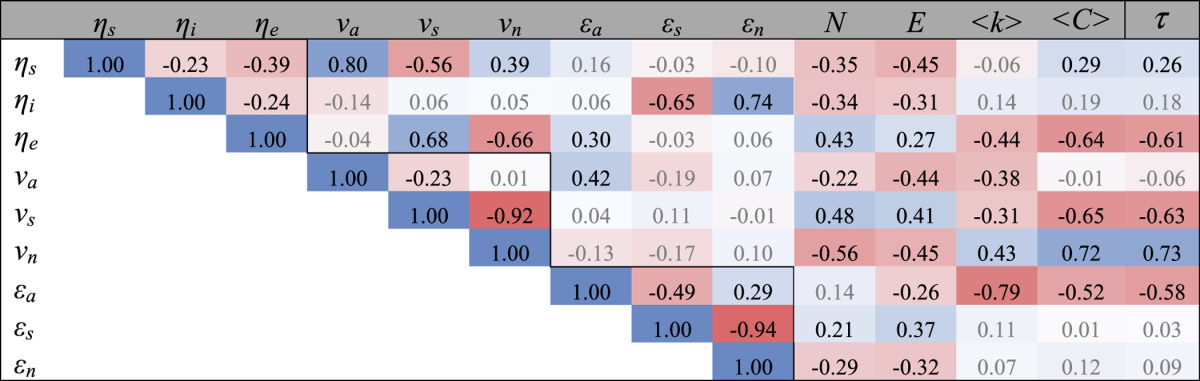
Spearman correlation coefficients between control parameters and basic network measures.

The table shows the total number of nodes and edges (*N* and *E*, respectively), the average degree <*k*>, average clustering coefficient <*C*>, and network transitivity *τ*. Table entries are colored according to their values (shades of blue for positive values and shades of red for negative); coefficients with a magnitude below 0.2 are written in light gray text. Black lines bracket intra-measure correlations (e.g. among *η*_*s*_, *η*_*e*_, and *η*_*i*_).
