# Supplementary material for: Correlations in the degeneracy of structurally controllable topologies for networks
Source: Sci Rep. 2017 Apr 12;7:46251. doi: 10.1038/srep46251 (PMC5388858; doi:10.1038/srep46251)
Supplement: Supplementary Information [file srep46251-s1.pdf]

Supplementary Information

Correlations in the degeneracy of structurally controllable topologies for networks

Colin Campbell, Steven Aucott, Justin Ruths, Derek Ruths, Katriona Shea, and Réka Albert

Additional Methods

Node-based assessment of MCT degeneracy

One can force a node to be directly controlled by temporarily removing it from the network and applying the Hopcroft-Karp algorithm to the modified network. However, for a network with N nodes and E edges the complexity of the Hopcroft-Karp algorithm is $O\left(EN^{1/2}\right)$. Thus the complexity of this approach is $O\left(EN^{3/2}\right)$. A second, more efficient method is to instead directly perturb the original MCT (Fig. S1). If n_i is added to N_d, then $|N_d|$ will increase by 1 unless a second node $m \in N_d$ can be removed from N_d as a result. In other words, the control signal that was being used to indirectly control node n_i must be re-routed to some node $m \in N_d$ such that it becomes indirectly controlled. Identifying a viable node m involves searching for paths that iteratively walk up matched edges and down unmatched edges (Fig. S1). If such a path terminates at a node $m \in N_d$, then node n_i can be directly controlled without increasing the size of N_d; that is, $n_i \in V_s$. Conversely, if no such path exists, then no MCT directly controls node n_i; that is, $n_i \in V_n$. The complexity of this approach is $O\left(EN\right)$ [1].

Edge-based control classification

Consider first the case of an edge $l_{ji} \in L_c$. The immediate effect of setting $l_{ji} \notin L_c$ is that node i must become directly controlled, as it is no longer a recipient of a control signal; that is, $|N_d|$ increases by 1. We wish to modify the control topology to reduce $|N_d|$ to its original value. To do this either node i must be indirectly controlled via a different incoming edge or some other node $m \in N_d$ must become indirectly controlled by virtue of the fact that node j may now send a control signal to some other downstream node. If either of these modifications are possible, $l_{ji} \in E_s$; otherwise $l_{ji} \in E_a$. Determining whether or not such paths exist involves searching for alternating paths on the network (Fig. S2).

Now consider the case of an edge $l_{ji} \notin L_c$. First suppose that node i is directly controlled. Because it has an upstream node j this configuration is only possible if node j has some other downstream node k such that the edge l_{jk} is on a control path. In this case l_{ji} may be set on a control path if l_{jk} is removed from its control path (Fig. S3a). Now if node i is indirectly controlled but $l_{ji} \notin L_c$, then there exists an edge $l_{mi} \in L_c$. If this is the case and node j has no outgoing edges that are on a control path, then we can similarly place l_{ji} on a control path by removing l_{mi} from its control path (Fig. S3b). The general condition of interest, then, is the case where node j has an outgoing edge on a control path, l_{jk}, and node i has an incoming edge on a control path, l_{mi} (Fig. S3c).

In this case, forcing l_{ji} to be on a control path requires removing both l_{jk} and l_{mi} from their control paths; as a result, if the control topology is not changed then node k must be directly controlled (i.e., $|N_d|$ will increase by 1; Fig. S3c). As above, two general strategies exist for changing the control topology to compensate: the control path originating at node m may be redirected to some other directly controlled node (thereby making it indirectly controlled), or node k may be indirectly controlled via some incoming edge other than l_{jk}. If either compensatory change

is possible, then $l_{ji} \in E_s$; otherwise $l_{ji} \in E_n$. The existence of viable changes (or the lack thereof) involves searching for alternating paths on the network (Fig. S3d-e).

Because a single search for alternating paths is of complexity $O(E)$ and we analyze every edge in this way, the overall complexity of this approach is $O(E^2)$, making the analysis of very large networks prohibitive with this approach.

Analysis of the T-LGL Leukemia network

We use the 60-node network of Saadatpour et al.[2] and remove the nodes "cytoskeleton signaling" and "proliferation", which are sink nodes that have no bearing on dynamics relevant to apoptosis. We additionally remove the node p27, which only affects proliferation. The subsequent network has 57 nodes and 141 edges. As noted in the main text, the literature indicates that control of three source nodes can lead to a leukemic state, and in this leukemic state control of any one among 18 of the remaining nodes can lead to apoptosis according to at least two of: experiment, simulation, and topological analysis. Hereafter we refer to these as the *key nodes*.

According to the metrics introduced in this report, 6 nodes are critical (i.e., they are sources), 16 are intermittent, and the remaining 35 are redundant. Three of the key nodes are critical and 6 are intermittent. The incomplete representation of critical and intermittent nodes is probably due to the specific setting (induced apoptosis of leukemic cells) studied by prior work. Key components of signaling cascades are often defined in terms of their role in modulating a signal as it propagates from source nodes to sink nodes. Thus, particularly for biological signaling networks we expect the edge-based metrics to offer more meaningful insight to the network.

In Table S2 we summarize the edge-based properties for the 18 key nodes that are not source nodes. In addition to the number and type of edges connected to (coming in or going out of) each key node, we provide two summary statistics for the overabundance of the edges connected to each node in each category. The *overabundance* is defined to be the difference between the number of edges in a category connected to a given node and the expected number under the assumption that the edges types are distributed randomly among all edges in the network. Because the expected number of edges is generally not an integer, in the *overabundance (linear)* column we first round the expected number of edges to the nearest integer. In both cases the A and S columns tend to be positive (highlighted in green) and the N column is not, suggesting that the 18 key nodes are essential regulators according to the edge-based control degeneracy metrics introduced in this report.

References

1. Jia, T. *et al.* Emergence of bimodality in controlling complex networks. *Nat. Commun.* **4,** (2013).

2. Saadatpour, A. *et al.* Dynamical and Structural Analysis of a T Cell Survival Network Identifies Novel Candidate Therapeutic Targets for Large Granular Lymphocyte Leukemia. *PLoS Comput Biol* **7,** e1002267 (2011).

3. Ruths, J. & Ruths, D. Control Profiles of Complex Networks. *Science* **343,** 1373–1376 (2014).

Table S1. The 58 empirical networks analyzed in this study. A superscript † indicates the edges of the network have been reversed in this analysis (see [3]).

Type	Name	N	Source
Neural	*C. Elegans*	1	http://toreopsahl.com/datasets
	Macaque	3	http://cocomac.g-node.org/<br>https://sites.google.com/site/bctnet/datasets<br>http://www.biological-networks.org/
Social	Email-EU	1	http://snap.stanford.edu/data/
	Intra-Organizational†	4	http://toreopsahl.com/datasets/
	Physician†	1	http://moreno.ss.uci.edu/data.html<br>*friendship network*
Corporate ownership	Corporate ownsership	1	http://vlado.fmf.uni-lj.si/pub/networks/data/econ/Eva/Eva.htm
Messaging	UC-Irvine	2	http://toreopsahl.com/datasets/
p2p	Gnutella	9	http://snap.stanford.edu/data/
Social influence	Physician†	2	http://moreno.ss.uci.edu/data.html
	Teacher-student	1	http://moreno.ss.uci.edu/data.html
	Wikipedia	1	http://snap.stanford.edu/data<br>*wiki-Talk*
transcription	*E. coli*	1	http://www.weizmann.ac.il/mcb/UriAlon/
	Yeast	1	http://www.weizmann.ac.il/mcb/UriAlon/
airport	airports	2	http://toreopsahl.com/datasets/
	airports-500	1	http://www.biological-networks.org/
Autonomous systems	autonomous	1	http://snap.stanford.edu/data/<br>*Nov. 5, 2007*
Electronic circuits	ISCA89	3	http://www.pld.ttu.ee/~maksim/benchmarks/<br>*s208, s420, s838*
Food webs	Food webs	22	http://vlado.fmf.uni-lj.si/pub/networks/data/bio/foodweb/foodweb.htm
blog	Political blog†	1	http://www-personal.umich.edu/~mejn/netdata/

Table S2. Edge-based control properties of the T-LGL signaling network. Key nodes that are not source nodes are grouped according to which of (1) experimental work, (2) simulation, and (3) topological analysis indicate that the node can induce apoptosis of leukemic cells if it is controlled. The number of edges connected to these nodes that are *always*, *sometimes*, and *never* on a control path are denoted by A, S, and N, respectively. The difference between the number of edges of each type connected to a node and the expected number are shown according to two metrics (see text). Positive overabundance (indicating the node has more edges of the given type than expected by random chance) is highlighted in green.

			edges				overabundance			overabundance (linear)		
	**node**	**node type**	**total**	**A**	**S**	**N**	**A**	**S**	**N**	**A**	**S**	**N**
**Expt., Sim., & Analysis**	S1P	R	6	1	2	3	0.26	0.07	-0.33	0	0	0
	PDGFR	R	6	1	2	3	0.26	0.07	-0.33	0	0	0
	SPHK1	I	2	0	2	0	-0.25	1.36	-1.11	0	1	-1
	Ceramide	R	4	1	2	1	0.50	0.72	-1.22	1	1	-1
	MEK	I	2	1	1	0	0.75	0.36	-1.11	1	0	-1
	ERK	R	5	1	3	1	0.38	1.39	-1.77	0	1	-2
	PI3K	I	7		4	3	-0.87	1.75	-0.88	-1	2	-1
	RAS	R	6	1	2	3	0.26	0.07	-0.33	0	0	0
**Expt. & Sim.**	STAT3	I	7	0	5	2	-0.87	2.75	-1.88	-1	3	-2
	MCL1	I	6	0	6	0	-0.74	4.07	-3.33	-1	4	-3
	NFKB	R	16	0	12	4	-1.99	6.86	-4.88	-2	7	-5
	JAK	R	8	0	5	3	-0.99	2.43	-1.44	-1	2	-1
**Sim. & Analysis**	DISC	R	9	0	4	5	-1.12	1.11	0.01	-1	1	0
	IL2RBT	R	3	1	2	0	0.63	1.04	-1.66	1	1	-2
	IL2RB	R	8	1	2	5	0.01	-0.57	0.56	0	-1	1
	sFas	R	3	1	1	1	0.63	0.04	-0.66	1	0	-1
	Fas	R	5	1	2	2	0.38	0.39	-0.77	0	0	-1
	TBET	I	5	0	5	0	-0.62	3.39	-2.77	-1	3	-3

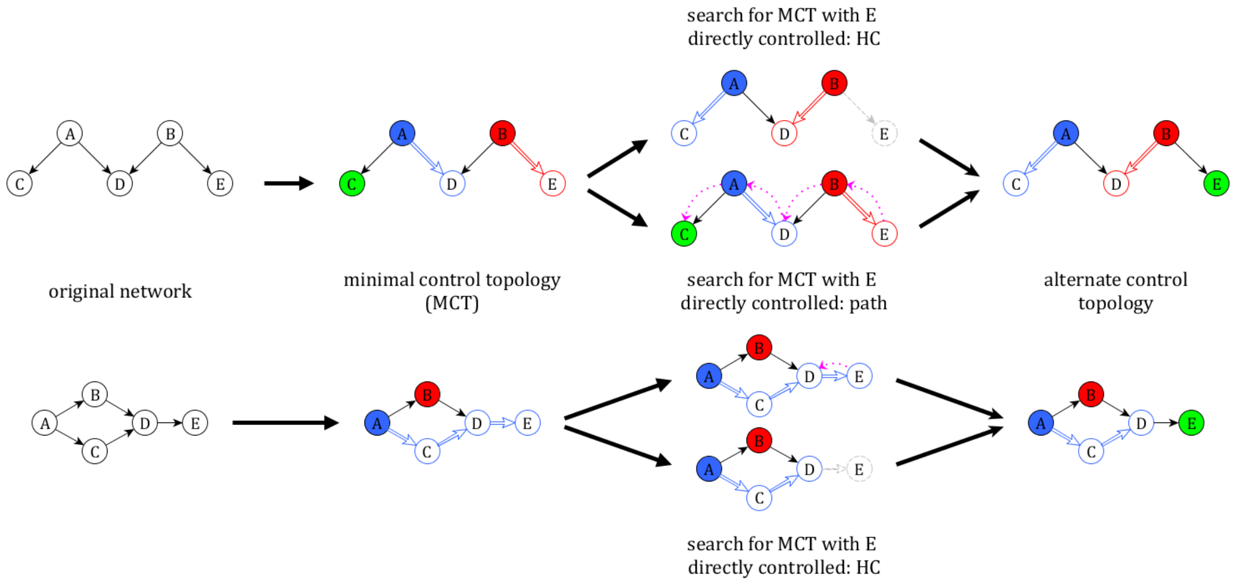

Fig. S1. Methods for determining if a node is *never* controlled or *sometimes* controlled across all minimal control topologies (MCTs). **(left column)** We consider two networks, shown with nodes and directed edges. **(second column)** For each network, we apply the Hopcroft-Karp algorithm to identify a MCT, where directly-controlled nodes are colored, the edges through which a control signal propagates are shown with colored outlines, and indirectly-controlled nodes are white with colored outlines. **(third column)** For both networks we seek to determine if a second minimal control topology exists such that node *E* is a directly controlled node (if such a control topology exists, node *E* is *sometimes* a directly-controlled node in the set of all MCTs; otherwise it is *never* directly controlled in the set of all MCTs; see text). For both networks we show two approaches: removing node *E* and applying the Hopcroft-Karp algorithm a second time (outer diagrams) and applying a directed walk up matched edges and down unmatched edges, until walking down to a directly controlled node (inner diagrams). These walks are shown with curved, dotted lines. In the case of the top network, such a path E->B->D->A->C is identified; in the case of the bottom network, the path unsuccessfully terminates after E->D. **(right column)** In the case of the top network, both the alternate and original control topologies have three directly-controlled nodes; node *E* is therefore *sometimes* directly controlled. The alternate control topology in the case of the bottom network also has three directly-controlled nodes, but the MCT has two. Therefore, in this case node *E* is *never* directly controlled among all possible MCTs.

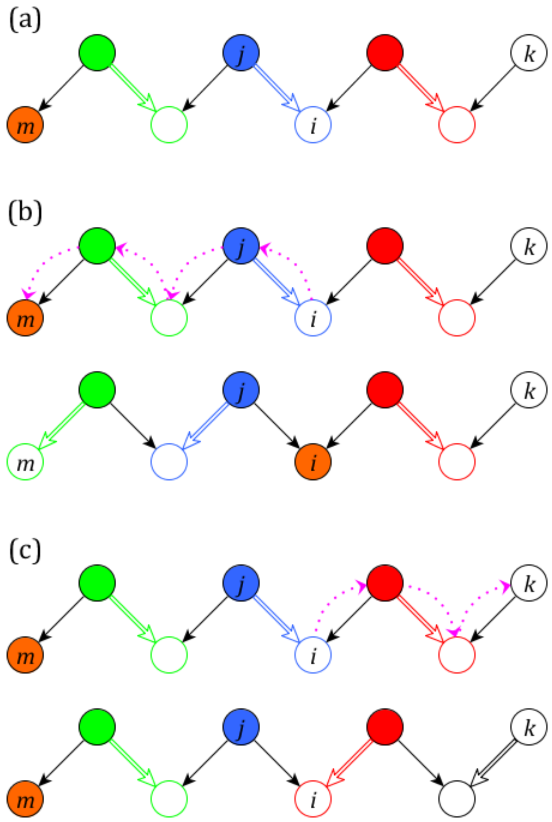

Fig. S2. Determining if an edge that is on a control path on one MCT is on a control path on *all* or only *some* MCTs. **(a)** An example component of an MCT, where directly-controlled nodes are colored, their control paths are represented by white arrows with colored outlines, and indirectly-controlled nodes are white with colored outlines. All nodes shown may in principle have additional connections. Note that node *k* may be either directly controlled or indirectly controlled, but we here assume it is *not* the source of an edge that is on a control path. **(b)** If we force the edge *j*→*i* to not be on a control path, we can search for paths that start at node *i*, then walk up control path edges and down regular edges, until the path walks down to a directly controlled node. In this example such a path (shown with dotted curved edges on the top panel) terminates at node *m*. The net effect is to exchange nodes *i* and *m* as directly vs. indirectly controlled. Thus an alternative MCT exists where node *i* is directly controlled but node *m* is not (bottom panel), and the edge *j*→*i* is *sometimes* on a control path in MCTs. **(c)** Alternatively, if we force the edge *j*→*i* to not be on a control path, we can search for paths that start at node *i*, walk up regular edges and down control path edges, until the path walks up to a node that does not have an outgoing control path edge. In this example such a path (shown with dotted curved edges on the top panel) terminates at node *k*. Thus an alternative MCT exists where node *i* is indirectly controlled via some node other than *j* (bottom panel), and the edge *j*→*i* is *sometimes* on a control path in MCTs. If no nodes matching the description of *m* or *k* exist, then edge *j*→*i* is *always* on a control path in MCTs.

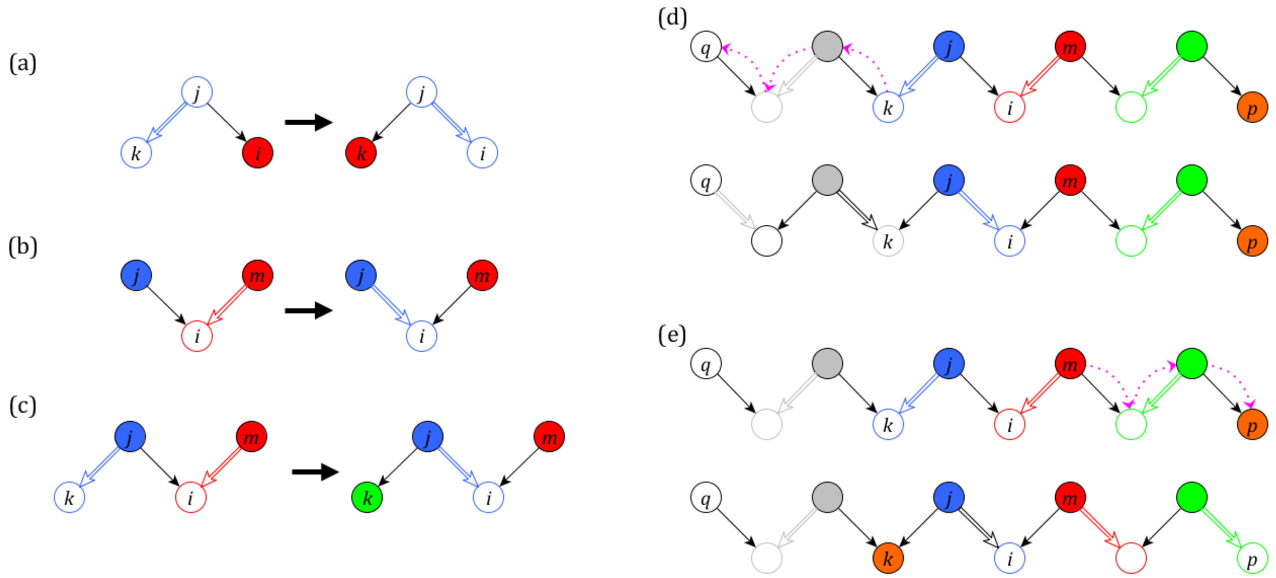

Fig. S3. Determining if an edge that is not on a control path in one MCT is *never* on a control path or *sometimes* on a control path across all MCTs. Coloring follows that of Figure 5. We seek to determine if it is possible to set the edge $j \rightarrow i$ to be on a control path given an MCT where it is not. **(a, left)** If i is directly controlled then there exists an edge $j \rightarrow k$ that is on a control path (otherwise $j \rightarrow k$ could be on a control path and i would be indirectly controlled, thereby reducing $|N_d|$ by 1, which is not possible since a MCT by definition has the minimal value of $|N_d|$). **(a, right)** Setting the edge $j \rightarrow i$ to be on a control path has no effect on $|N_d|$, as node i is now indirectly controlled but node k is directly controlled. **(b)** If node i is *not* directly controlled, it is indirectly controlled via some node m. If node j additionally has no outgoing edges on a control path, then the control of node i may be transferred from node m to node j without affecting $|N_d|$. **(c)** If neither situation (a) nor (b) applies, then node i is indirectly controlled via some node m and node j indirectly controls some node k. Forcing edge $j \rightarrow i$ to be controlled necessarily forces the edges $j \rightarrow k$ and $m \rightarrow i$ to become regular edges (i.e, not on a control path), which forces node k to be directly controlled and increases $|N_d|$ unless compensatory changes to the control topology are made. In panels **(d, e)** we show the MCT topology of (c) embedded in a broader MCT. All nodes shown may in principle have additional connections. Note that node q may be either directly controlled or indirectly controlled, but we here assume it is *not* the source of an edge that is on a control path. **(d, top)** If we force the edge $j \rightarrow i$ to be on a control path, we can search for paths that start at node k, then walk up regular edges and down control path edges, until the path walks up to a node with no outgoing edges on a control path. In this example such a path (shown with dotted curved edges) terminates at node q. **(d, bottom)** Thus at least one alternative MCT exists where edge $j \rightarrow i$ is on a control path and overall the edge is categorized as *sometimes* existing on a control path in MCTs. **(e, top)** Alternatively, if we force the edge $j \rightarrow i$ to be on a control path, we can search for paths that start at node m, then walk down regular edges and up control path edges, until the path walks down to a directly controlled node. In this example such a path (shown with dotted curved edges) terminates at node p; the net effect is to exchange nodes k and q as directly vs. indirectly controlled. **(e, bottom)** Thus at least one alternative MCT exists where edge $j \rightarrow i$ is on a control path and overall the edge is categorized as *sometimes* existing on a control path in MCTs. If no nodes matching the description of q or p exist, then edge $j \rightarrow i$ is *never* on a control path in MCTs.
